# Eicosapentaenoic acid limits the more rapid oxidation of lipoprotein(a) compared with other apolipoprotein B particles

**DOI:** 10.1093/cvr/cvaf144

**Published:** 2025-08-25

**Authors:** Samuel C R Sherratt, Peter Libby, Richard L Dunbar, Deepak L Bhatt, R Preston Mason

**Affiliations:** Mount Sinai Fuster Heart Hospital, Icahn School of Medicine at Mount Sinai, 1 Gustave L. Levy Pl, New York, NY 10029, USA; Elucida Research, 100 Cummings Center, Suite 135L, Beverly, MA 01915, USA; Department of Medicine, Cardiovascular Division, Brigham and Women’s Hospital, Harvard Medical School, 75 Francis St, Boston, MA 02115, USA; Amarin Pharma, Inc., 440 Route 22 East, Suite 300, Bridgewater, NJ 08807, USA; Mount Sinai Fuster Heart Hospital, Icahn School of Medicine at Mount Sinai, 1 Gustave L. Levy Pl, New York, NY 10029, USA; Elucida Research, 100 Cummings Center, Suite 135L, Beverly, MA 01915, USA; Department of Medicine, Cardiovascular Division, Brigham and Women’s Hospital, Harvard Medical School, 75 Francis St, Boston, MA 02115, USA

**Keywords:** Lipoprotein(a), Oxidation, Eicosapentaenoic acid

## Abstract

**Aims:**

Elevated lipoprotein(a) [Lp(a)] levels increase cardiovascular (CV) risk. Lp(a) contains oxidized phospholipids that may promote lipid oxidation more than other lipoproteins. The highly unsaturated omega-3 fatty acid eicosapentaenoic acid (EPA) has multiple double bonds that can trap free radicals in resonance structures. Purified ethyl-EPA reduced CV events in high-risk patients with elevated Lp(a) despite Lp(a)-associated risk elevation. Since Lp(a) is enriched in oxidized lipids, we hypothesized that Lp(a)-enriched plasma undergoes more rapid oxidation than other apolipoprotein B (ApoB)-containing particles and that EPA limits oxidation of Lp(a)-enriched plasma more effectively than less-unsaturated fatty acids or other lipid-lowering treatments. This property could limit the cellular stress responses in endothelial cells (ECs).

**Methods and results:**

Lp(a) was enriched to >50% total ApoB content to resemble an Lp(a)-associated ‘high-risk’ phenotype and compared with matching levels of small-dense LDL (sdLDL) and very-low-density lipoprotein by isopycnic centrifugation. Samples were then incubated with EPA (50 µM) or equivolume vehicle at 37°C for 30 min. Oxidation was initiated with copper sulfate and monitored by malondialdehyde formation. We also subjected EPA to oxidation before measuring its antioxidant activity when compared with other long chain, less saturated fatty acids and lipid-lowering agents. Human umbilical vein ECs (HUVECs) were incubated with Lp(a)-enriched plasma following oxidation in the absence and presence of EPA. Cell lysate samples were then analysed by global liquid chromatography-mass spectroscopy (LC/MS)–based proteomics for significant changes in protein expression (>1-fold). Lp(a)-enriched plasma contained the highest baseline oxidized lipid (*P* < 0.05) and underwent the most rapid oxidation. EPA, but neither the less-unsaturated fatty acids nor lipid agents attenuated oxidation of each fraction through 4 h (*P* < 0.01). Oxidized EPA had diminished antioxidant capacity corresponding to the extent of oxidation. Attenuation of Lp(a) oxidation with EPA also mitigated pro-inflammatory and cellular stress response changes in protein expression.

**Conclusions:**

Lp(a)-enriched plasma underwent more rapid oxidation than other ApoB-containing lipoproteins and promoted inflammatory changes in EC protein expression, a process attenuated by EPA. This action may contribute to reduced CV risk by EPA in those with elevated Lp(a) levels.

## Graphical Abstract

**Graphical Abstract:**
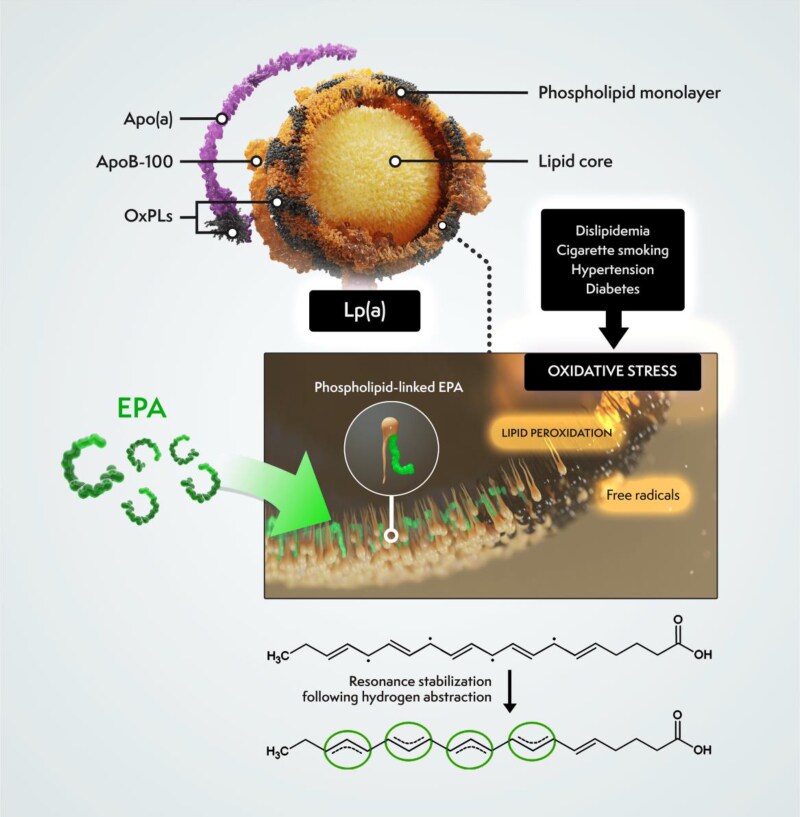
Proposed mechanism of antioxidant activity of eicosapentaenoic acid (EPA) in Lp(a). Lp(a)-enriched lipoprotein fractions undergo more rapid lipid oxidation compared with other apolipoprotein B (ApoB)-containing lipoproteins, possibly due to oxidized phospholipids (oxPLs) attached to its unique apo(a) and in the phospholipid monolayer surrounding the insoluble lipid-rich core. The highly unsaturated fatty acid EPA exhibits significant dose- and time-dependent antioxidant activity in Lp(a). This action may arise from the five double bonds along the acyl chain allow for free radical resonance stabilization and may contribute to significant cardiovascular event reduction by highly purified EPA [delivered as icosapent ethyl (IPE)], particularly patients with elevated Lp(a) (REDUCE-IT).

**Time of primary review: 30 days**

**See the editorial comment for this article ‘Beyond triglycerides lowering: rethinking the role of eicosapentaenoic acid in lipoprotein biology’, by A. Tirandi *et al*., https://doi.org/10.1093/cvr/cvaf175.**

## 1. Introduction

Observational and epidemiologic investigations indicate an independent, causal association between elevated lipoprotein(a) [Lp(a)] levels and risk for atherosclerotic cardiovascular (CV) disease (ASCVD) as well as aortic valve stenosis/calcification.^1^ An individual’s Lp(a) levels are largely (>90%) genetically determined based on the number of KIV_2_ repeats found within the *LPA* gene, and therefore lifestyle modification has little to no effect on Lp(a) levels.^2^ Atherogenic mechanisms attributed to Lp(a) include increased inflammation linked to oxidized phospholipid (oxPL) bound to the kringle-like domain KIV_10_ on apolipoprotein(a) [apo(a)] and throughout the phospholipid monolayer.^3^ Clinical investigations show a direct relationship between oxPL levels (expressed as oxPL/ApoB ratio) and both coronary artery disease and aortic valve calcification.^4,5^ Lp(a)-enriched plasma may have enhanced susceptibility to oxidation due to oxPL, which may serve as ‘seeding sites’ for free radicals promoting propagation of lipid oxidation. Similarly, small-dense LDL (sdLDL) may promote atherogenesis due to prolonged retention in the intima and greater susceptibility to oxidative modification that may be due to its lower radius of curvature compared to larger LDL and triglyceride-rich particles [e.g. very-low-density lipoprotein (VLDL)]. Lipoprotein oxidation fosters foam cell formation and increased arterial lipid burden, and oxidized LDL associates with higher risk of ASCVD events in patients with coronary disease.^6^ Levels of oxidized Lp(a) predicted CV events in Type 2 diabetes better than non-oxidized Lp(a).^7^ These observations inspire the hypothesis that therapeutic interventions that reduce the oxidation of Lp(a) limit its atherogenicity independent of changes in concentration.

A *post-hoc* analysis of the Reduction of Cardiovascular Events with Icosapent Ethyl–Intervention Trial (REDUCE-IT) showed consistent CV risk reduction in patients with or without elevated Lp(a) at baseline treated with 4 g/day icosapent ethyl (IPE), an ethyl ester of the omega-3 fatty acid (n-3FA) eicosapentaenoic acid (EPA; 20:5), despite the higher risk associated with elevated Lp(a) and no change in Lp(a) levels with IPE treatment.^8,9^ The mechanism may relate to free radical scavenging activity of EPA at higher achieved concentrations among patients randomized to IPE. We have previously shown that EPA effectively reduces oxidation of ApoB-containing particles *in vitro* compared with other long chain fatty acids tested at pharmacologic concentrations.^10,11^ In particular, long chain fatty acids without multiple double bonds (e.g. stearic acid [18:0], arachidic acid [ArA, 20:0]) or other polyunsaturated fatty acids of varying acyl chain length and double bond distribution [e.g. docosahexaenoic acid (DHA) (22:6), arachidonic acid (20:4)] did not share this activity of EPA, consistent with less stable interactions with neighboring lipid molecules.^12^ This observation suggests that the highly unsaturated nature of EPA combined with its acyl chain length (C20) facilitates resonance stabilization of free radicals following low energy hydrogen abstraction.

We therefore tested the hypothesis that Lp(a)-enriched lipoprotein samples that simulate an elevated ASCVD risk phenotype^13^ (>50% of total ApoB) undergo more rapid oxidation compared with other enriched lipoproteins, specifically sdLDL, non-modified LDL, and VLDL. Our molecular hypothesis was that pharmacologic concentrations of EPA exhibit antioxidant activity in these particles because its highly unsaturated composition combined with optimal chain length foster free radical scavenging. Accordingly, we contrasted EPA with other C20 fatty acids lacking multiple double bonds. We also subjected EPA and the n-3FA alpha-linolenic acid (ALA) to conditions shown to cause systematic oxidative modification along the acyl chain, including loss of alkene bonds and chain breakage,^14,15^ to test whether such alterations compromise the antioxidant capacity of n-3FAs.We also compared the antioxidant activity of EPA with other small molecule lipid-lowering agents with different physico-chemical properties. Finally, we measured the effects of Lp(a) in the absence and presence of EPA and exposed to oxidative conditions on protein expression in vascular endothelial cells (ECs).

## 2. Methods

### 2.1 Material isolation and preparation of lipoprotein fractions

All lipoprotein fractions were tested for purity and combined with LDL to match the Lp(a) enrichment (66%). Lp(a) fractions were pooled from multiple (*n* = 2–3) healthy donors with elevated Lp(a) levels. In brief, plasma containing Lp(a) was adjusted to a density of 1.066 using potassium bromide as previously described.^16^ It was then subjected to 185 000 *g* at 5°C for 24 h in an ultra-centrifuge. The denser fraction was collected and adjusted to 1.082 using potassium bromide. The second spin was for 48 h at 185 000 *g* and 5°C. Multiple fractions were collected and evaluated using an agarose gel. The well-defined bands of Lp(a) were dialysed and concentrated. We then prepared matching 66% sdLDL-and VLDL-enriched samples. Non-modified LDL (i.e. 100%) was also analysed in parallel. EPA, niacin, and colorimetric reagents were purchased from Sigma-Aldrich (St. Louis, MO, USA). Gadoleic acid (GA, 20:1), ArA (20:0), ALA, rosuvastatin, probucol, and fenofibric acid were purchased from Cayman Chemicals (Ann Arbor, MI, USA). All reagents were solubilized in ethanol and stored at −20°C prior to testing; thus, all vehicle-treated controls contained equivolume ethanol delivered in parallel with drug treatment. Primary human umbilical vein ECs (HUVECs, PCS-100-013™) were purchased from ATCC (Manassas, VA, USA) and grown in the recommended complete endothelial growth medium (Vascular Cell Basal Media, ATCC PCS-100-030™) supplemented with Endothelial Cell Growth Kit (ATCC PCS-100-040™), 5% foetal bovine serum, and maintained at 37°C in a 95% air/5% CO_2_ humidified incubator.

### 2.2 Lipoprotein oxidation

Oxidation of the various lipoprotein fractions used previously described methods.^10,11,17^ Briefly, 50 µg/mL extracts from each lipoprotein fraction were incubated at 37°C for 30 min in the presence of the test treatment or equivolume vehicle and then subjected to copper sulfate–induced oxidation (20 µM). At multiple time points through 4 h, aliquots from each test sample were removed and monitored by formation of malondialdehyde (MDA) as previously described.^10^ We also measured formation of primary lipid hydroperoxides (LOOHs) from oxidized Lp(a) and LDL as previously described to confirm oxidation of the particles.^10,18^

We first compared the rate of oxidation of each lipoprotein fraction in the absence of drug treatment. This included Lp(a)-enriched [Lp(a) + LDL], sdLDL-enriched (sdLDL + LDL), non-modified LDL (100% LDL), and VLDL-enriched (VLDL + LDL) samples. Due to the significant differences in lipid content of each lipoprotein, rate of oxidation was expressed as the percentage of total lipid oxidation at each time point through 2 h. This property was defined as the peak measured MDA value obtained over the course of the analysis.

We then analysed the dose-dependent effects of EPA across a range of physiological concentrations (10–100 µM) in Lp(a) to determine the maximum active concentration (50 µM). The activity of EPA (50 µM) was then compared with equimolar concentrations of GA and ArA as well as fenofibric acid, rosuvastatin, niacin, and the lipoprotein-penetrant scavenging antioxidant probucol (10 µM each) in each lipoprotein fraction.

### 2.3 Oxidation of EPA

To understand whether oxidative damage to EPA impairs its antioxidant activity, we exposed vials containing 10 mg pure EPA to ambient laboratory conditions, thus allowing it to degrade over time (0–10 days). We then monitored primary (LOOH) and secondary (MDA) lipid oxidation products in these samples to confirm degradation of the EPA acyl chains. Finally, these oxidized EPA (oxEPA) samples were evaluated for antioxidant activity in isolated human LDL.

Starting with the 10 day oxidation sample, one new ampule of pure EPA was opened at each time point and exposed to ambient conditions. The temperature was held constant in the lab each day, and the samples were placed in one open top container to allow uniform exposure to ambient light.

Just before the LDL oxidation analysis, samples were assayed for LOOH and MDA as previously described.^10^ To perform these assays, a portion of each oxEPA sample was removed via a glass Pasteur pipette and weighed. These aliquots were then solubilized in ethanol prior to LOOH and MDA measurements. The antioxidant capacity of each oxEPA sample (50 µM) was assessed in isolated human LDL as previously described in section 2.2. We then repeated this analysis with ALA to assess whether changes in acyl chain length or degree of unsaturation affected oxidation rate or loss of antioxidant capacity in LDL.

### 2.4 Proteomic analysis of oxidized Lp(a)-treated HUVECs

To further explore the potential pro-atherogenic effects of oxidized Lp(a), we treated HUVECs with Lp(a) (150 µg/mL) exposed to EPA (50 µM) or equivolume ethanol vehicle and oxidized with copper sulfate for 2 h as described in section 2.2. Cells were incubated with Lp(a) + ethanol vehicle [Lp(a) + veh] or Lp(a) + EPA for 8 h, after which protein content was extracted and prepared for proteomic analysis. We used tandem mass tag labeling coupled to LC-MS–based proteomic analysis as previously described.^19^

### 2.5 Statistical analyses

Raw protein intensity mass spectrometry data and false discovery rate–adjusted *P*-values were analysed and calculated using the Differential Enrichment of Proteomics bioinformatic package v.1.26.0. Significantly modulated proteins were defined as those with a between group fold change >1.0 and adjusted *P*-value <0.05. This program also generated the heatmaps, principal component analysis plot, and bar graphs of normalized raw intensity data for proteins across treatment groups.

For the lipoprotein oxidation experiments, statistical comparisons were analysed by the Tukey–Kramer multiple comparisons test for comparison of multiple groups or Student’s *t*-test for comparisons between two groups. Normality was assessed by Kolmogorov–Smirnov test. The relationship between duration of EPA oxidation and antioxidant activity in LDL was assessed using a Spearman’s rank correlation coefficient (*ρ*). Statistical tests were carried out using GraphPad InStat 3 or Stata, and graphs were created using Origin v8.6 or Stata.

## 3. Results

### 3.1 Lipoprotein oxidation rates

**Figure 1:**
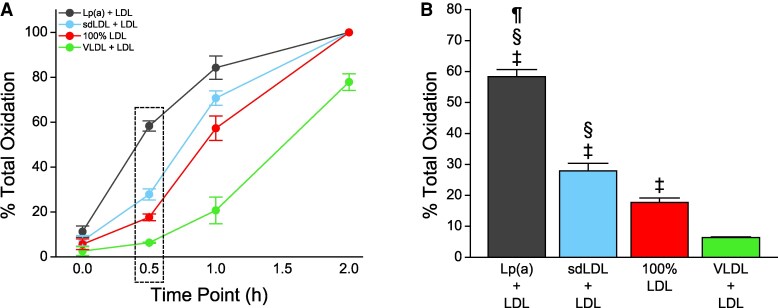
Lp(a)-enriched lipoprotein fractions undergo more rapid oxidation than other lipoprotein fractions. (*A*) Data expressed as per cent of total oxidation, which is defined as the peak measured MDA equivalent value obtained at a given time point. The per cent total oxidation therefore is calculated by dividing the MDA equivalents at a time point prior to 2 h by the peak MDA equivalents measured. (*B*) The 0.5 h time point of this analysis. Statistical indicators: ^‡^*P* < 0.001 vs. VLDL; ^§^*P* < 0.001 vs. 100% LDL; ^¶^*P* < 0.001 vs. sdLDL + LDL (Tukey–Kramer multiple comparisons test; overall ANOVA: *P* < 0.0001). Values are mean ± standard deviation (*N* = 6).

**Figure 2:**
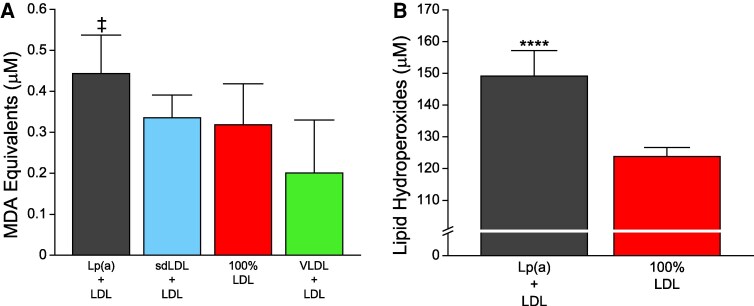
Lp(a) contains more oxidized lipid content than other fractions as measured by (*A*) secondary MDA and (*B*) primary LOOHs. Statistical indicators: (*A*) ^‡^*P* < 0.05 vs. sdLDL + LDL and VLDL + LDL. (*B*) ****P = 0.0068 vs. 100% LDL (Tukey–Kramer multiple comparisons test; overall ANOVA: *P* < 0.0001). Values are mean ± standard deviation (*N* = 3–6).

In this study, the peak MDA values for Lp(a), sdLDL, and LDL-enriched samples occurred at 2 h, whereas peak MDA values for VLDL-enriched samples occurred at 4 h. The per cent total oxidation was therefore calculated by dividing the MDA equivalents at time points prior to 2 h by the peak MDA equivalents measured at 2 or 4 h, respectively. We found that as early as 0.5 h, the Lp(a)-enriched fraction underwent increased oxidation compared with all other preparations (*Figure 1A*; see Supplementary material online, *Figure S1*). Analysing the rate of change of each fraction revealed the largest slope value at the 0.5 h time point attributable to the Lp(a)-enriched fraction (see Supplementary material online, *Figure S2A* and *B*). The earlier onset of oxidation seen in the Lp(a)-enriched samples at 30 and 60 min was sufficient for the area under the curve (AUC) to remain higher than the AUC of the other lipoproteins from 0 to 2 h (*P* < 0.001, Supplementary material online, *Figure S2C* and *D*), suggesting this is not a transient effect. Indeed, at 0.5 h, 58% of the total oxidizable lipid in the Lp(a)-enriched sample, compared with 28% in the sdLDL-enriched sample, 18% in the non-enriched LDL sample, and 6% in the VLDL-enriched sample (*Figure 1B*). Baseline measurements (0 h) showed Lp(a)-enriched samples contained elevated levels of oxidation products (e.g. MDA) compared with sdLDL (32%) and VLDL (120%) enrichments (*P* < 0.05) (*Figure 2A*). While the baseline levels of MDA in Lp(a)-enriched samples were 39% higher than non-enriched LDL, this did not reach statistical significance (*P* = 0.05). However, baseline levels of LOOH were 20% higher in Lp(a)-enriched samples than non-enriched LDL samples (*P* = 0.007) (*Figure 2B*). Together, these results indicate the suspected presence oxidized lipids in the Lp(a)-enriched sample prior to exogenous initiation of oxidation.

### 3.2 Effects of EPA on lipoprotein oxidation

**Figure 3:**
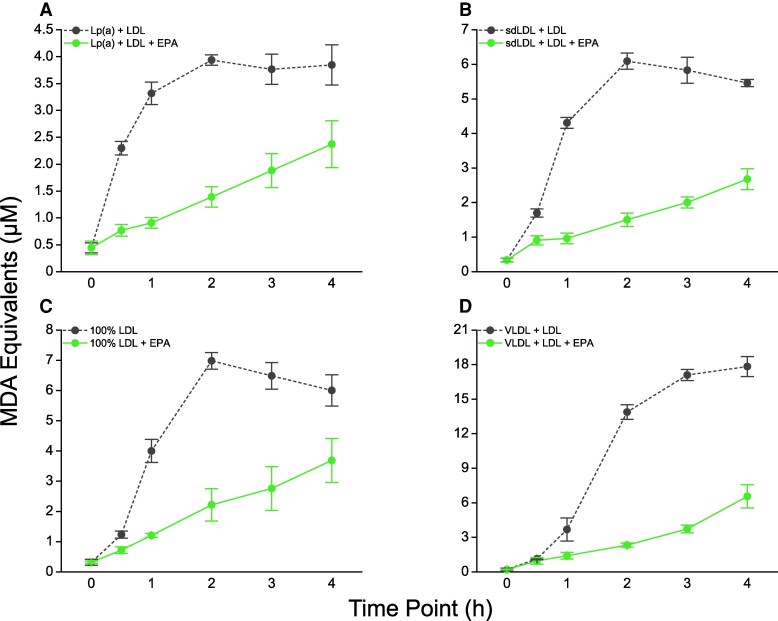
Time-dependent effects of EPA on oxidation of (*A*) Lp(a)-, (*B*) sdLDL-, (*C*) LDL-, and (*D*) VLDL-enriched lipoprotein fractions. At all time points from 1 through 4 h, EPA significantly reduced MDA formation from each lipoprotein fraction compared with the cognate vehicle treatment (*P* < 0.001; tested using unpaired, two-tailed Student’s *t*-test at each time point). Values are mean ± standard deviation (*N* = 6).

In each lipoprotein fraction, EPA exhibited dose- and time-dependent antioxidant activity over a range of pharmacological concentrations (see Supplementary material online, *Figure S3*). Except for VLDL-enriched samples, maximum activity was plateaued at 50 µM. Through 4 h, EPA 50 µM significantly attenuated oxidation of each fraction by ≥38% (*Figure 3A–D*). After 0.5 h, where the increased rate of oxidation in Lp(a)-enriched samples was first detected, EPA produced the largest reduction in MDA formation in Lp(a) (67%) compared with the other fractions. EPA also significantly inhibited MDA formation by >63% at the peak oxidation levels of each lipoprotein fraction. We separately compared the antioxidant effects of EPA and probucol in Lp(a)-enriched fractions using LOOH and MDA measurements and found that EPA significantly reduced both oxidation products compared with peak levels (both *P* < 0.001) and to a greater extent than probucol (see Supplementary material online, *Figure S4*).

### 3.3 Effects of oxidative damage on EPA activity

We hypothesized that the antioxidant activity of EPA arises from the multiple methylene-interrupted double bonds along the acyl chain, which both decrease the strength of C–H bonds along the acyl chain to allow for more efficient hydrogen abstraction by free radicals and provide resonance stabilization for the resulting carbon-centric radicals. Over time, EPA loses its antioxidant capacity as the double bonds can no longer effectively stabilize these radicals leading to acyl chain breakage.^14^ Over 10 days exposure to ambient conditions, pure EPA underwent consistent oxidative damage as evidenced by increased levels of LOOH and MDA, reaching peak levels after 5 days corresponding to 517 and 890% increases, respectively (see Supplementary material online, *Figures S5*–*S7*). Oxidation of the n-3FA ALA followed a similar pattern with LOOH and MDA levels consistently increasing from 0–5 days with a 267 and 230% increase, respectively, after 5 days (see Supplementary material online, *Figure S6*). As expected, there was a strong correlation between LOOH and MDA levels in EPA and ALA samples (*ρ* = 1, *P* = 0.0029 and *ρ* = 0.89, *P* = 0.0259, respectively, Supplementary material online, *Figure S7*).

**Figure 4:**
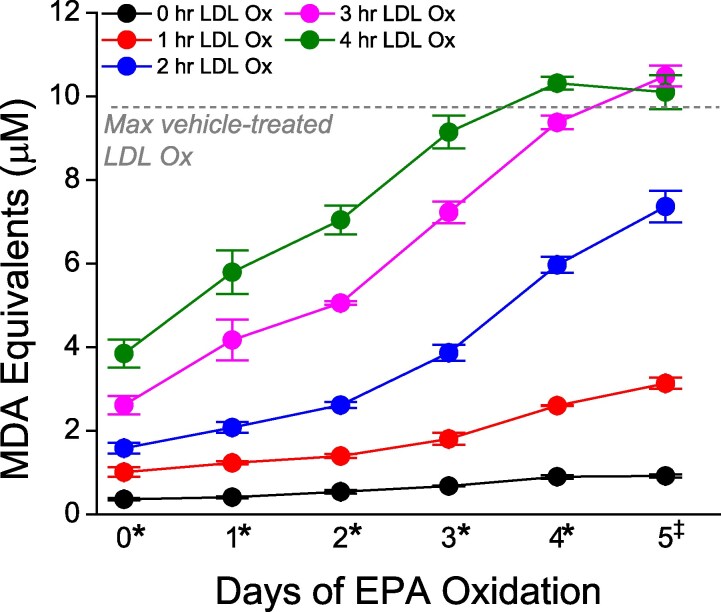
Effects of time-dependent oxidation of EPA on oxidation of human LDL—0–5 days EPA oxidation. Statistical analysis performed between different oxEPA samples across the duration of LDL oxidation. Statistical indicators: *P < 0.05 for all sample comparisons; ^‡^*P* < 0.05 for all sample comparisons except 3 and 4 h LDLox (Tukey–Kramer multiple comparisons test; overall ANOVA: *P* < 0.0001). Values are mean ± standard deviation (*N* = 3).

**Figure 5:**
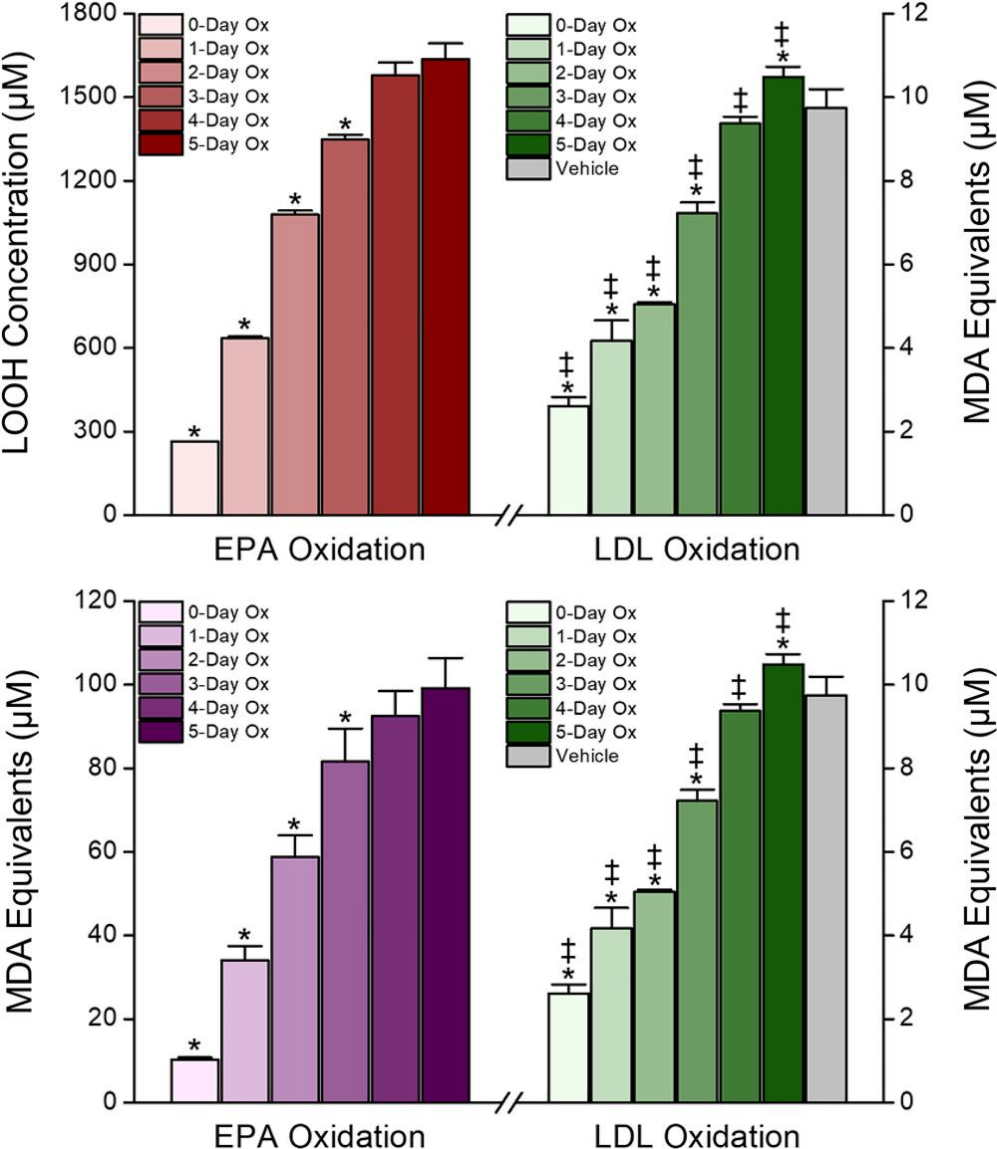
Correlation of 1° (LOOH) and 2° (MDA) oxidation products and antioxidant activity of oxEPA in human LDL through 3 h. Statistical indicators: TBARS Stats: *P < 0.05 vs. all other oxidation durations (Tukey–Kramer multiple comparisons test; overall ANOVA: *P* < 0.0001). LOOH Stats: *P < 0.01 vs. all other oxidation durations (Tukey–Kramer multiple comparisons test; overall ANOVA: *P* < 0.0001). LDL Ox Stats: *P < 0.05 vs. vehicle; ^‡^*P* < 0.05 vs. all other oxidation durations (Tukey–Kramer multiple comparisons test; overall ANOVA: *P* < 0.0001). Values are mean ± standard deviation (*N* = 3).

As the EPA samples became more oxidized, there was a concomitant decrease in antioxidant capacity as evidenced by the increased rate of LDL oxidation with these oxEPA samples (see Supplementary material online, *Figure S8*; *Figures 4* and *5*). In *Figure 4*, each line represents a different time point in the LDL oxidation assay through 4 h. As the exposure time of EPA to oxidative conditions increases (left to right along the *x*-axis), the MDA equivalents produced by LDL oxidation increased consistently in all samples except the native EPA, which sustained its antioxidant activity at each time point. After 4 h, the 0 day (58%) through 2 day (23%) oxEPA samples significantly reduced LDL oxidation compared with the vehicle control (*P* < 0.05), while the 3–10 day old EPA samples could not sustain an antioxidant effect. Further, 1 day of autoxidation more than halved the activity of the undamaged EPA, reducing activity by 51%. The correlation between EPA oxidation products and antioxidant capacity is shown side-by-side in *Figure 5* and Supplementary material online, *Figure S8*. We observed a similar, consistent loss of antioxidant capacity of ALA concomitant with increased oxidation products, though overall ALA was less active than EPA at each oxidation exposure (see Supplementary material online, *Figures S9* and *S10*).

### 3.4 Comparisons with less-unsaturated fatty acids and lipid-lowering agents

**Figure 6:**
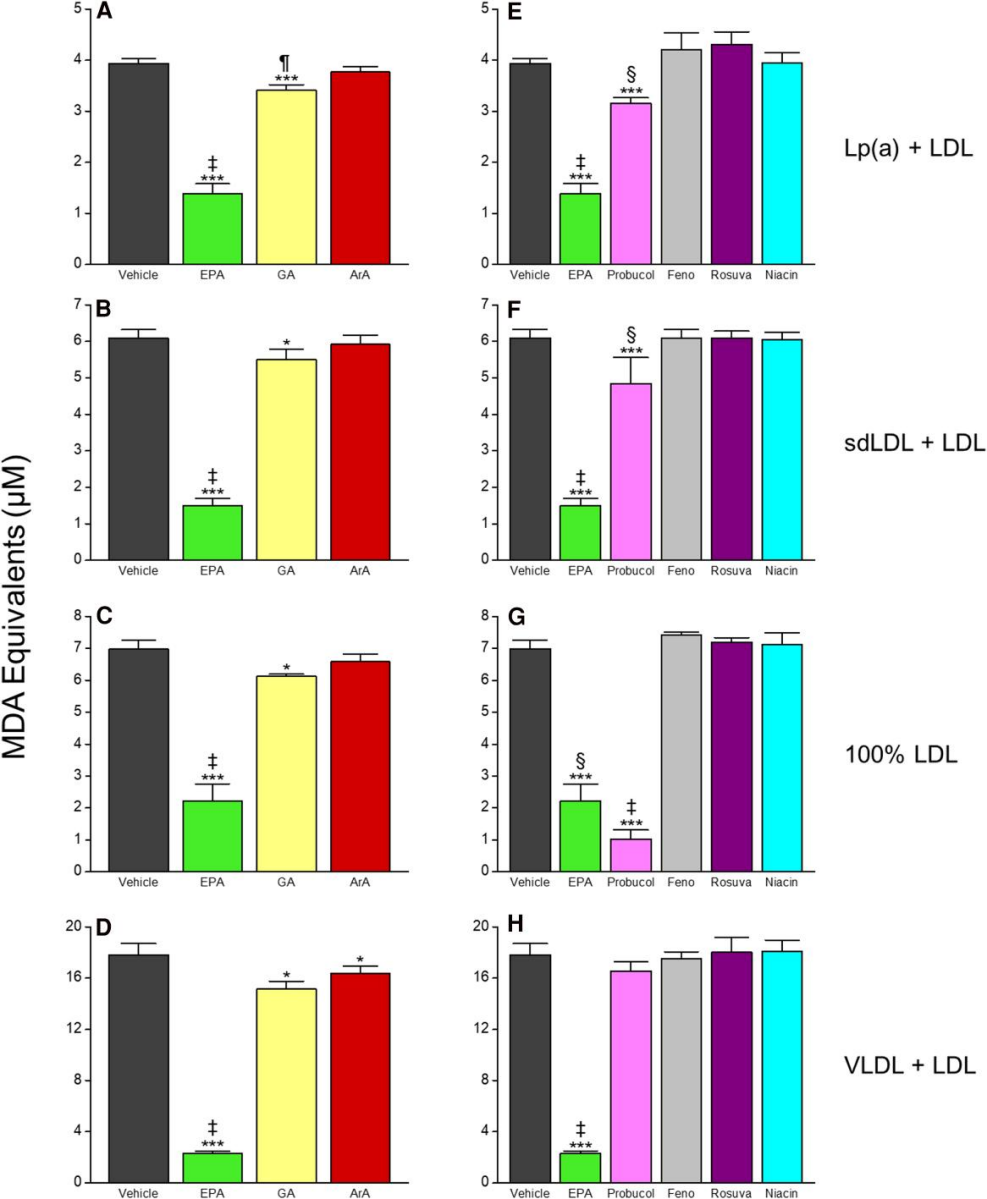
Effects of EPA vs. comparators on oxidation of Lp(a)-, sdLDL-, LDL-, and VLDL-enriched lipoprotein fractions. Statistical indicators: (*A*) ***P < 0.001 vs. vehicle; ^‡^*P* < 0.001 vs. GA and ArA; ^¶^*P* < 0.05 vs. ArA; (*B*) ***P < 0.001 vs. vehicle; *P < 0.05 vs. vehicle; ^‡^*P* < 0.001 vs. GA and ArA; (*C*) ***P < 0.001 vs. vehicle; *P < 0.05 vs. vehicle; ^‡^*P* < 0.001 vs. GA and ArA; (*D*) ***P < 0.001 vs. vehicle; *P < 0.05 vs. vehicle; ‡*P* < 0.001 vs. GA and ArA; (*E*) ***P < 0.001 vs. vehicle; ^‡^*P* < 0.001 vs. all treatments; ^§^*P* < 0.01 vs. Feno, Rosuva, and Niacin; (*F*) ***P < 0.001 vs. vehicle; ^‡^*P* < 0.001 vs. all treatments; ^§^*P* < 0.01 vs. Feno, Rosuva, and Niacin; (*G*) ***P < 0.001 vs. vehicle; ^‡^*P* < 0.01 vs. all treatments; ^§^*P* < 0.001 vs. Feno, Rosuva, and Niacin; (*H*) ***P < 0.001 vs. vehicle; ^‡^*P* < 0.001 vs. all treatments. All tests performed using Tukey–Kramer multiple comparisons test; overall ANOVA: *P* < 0.0001). Values are mean ± standard deviation (*N* = 3–6). ArA, arachidic acid; Feno, fenofibric acid; GA, gadoleic acid; Rosuva, rosuvastatin.

To affirm the structure-dependent activity of EPA, we then compared it with other C20 fatty acids with fewer (GA, 20:1) or no (ArA, 20:0) alkene double bonds (*Figure 6A–D*). Compared with the peak oxidation of each lipoprotein fraction, ArA did not significantly reduce MDA formation except a small, 8% change in VLDL + LDL fractions. GA, which is a monounsaturated fatty acid, did significantly attenuate MDA formation in each fraction (13%, 10%, 15%, 12% in Lp(a)-enriched, sdLDL-enriched, VLDL-enriched, and non-modified LDL, respectively all *P* < 0.05), although not to the same degree as EPA (65, 75, 87, 68%, respectively, in those same fractions, all *P* < 0.001). We then tested whether other lipid-lowering agents with different chemical structures would yield any antioxidant activity. Neither rosuvastatin, fenofibric acid, nor niacin significantly affected the oxidation rate of any lipoprotein fraction (*Figure 6E–H*). The lipophilic scavenging antioxidant probucol exhibited antioxidant activity in all fractions as expected though in general to a lesser extent than EPA: 20% reduction in Lp(a)-enriched (*P* < 0.001), 21% reduction in sdLDL-enriched (*P* < 0.001), 7% reduction in VLDL-enriched, and 85% reduction in non-modified LDL fractions (*P* < 0.001).

### 3.5 Effects of Lp(a) ± EPA on protein expression in HUVECs

**Figure 7:**
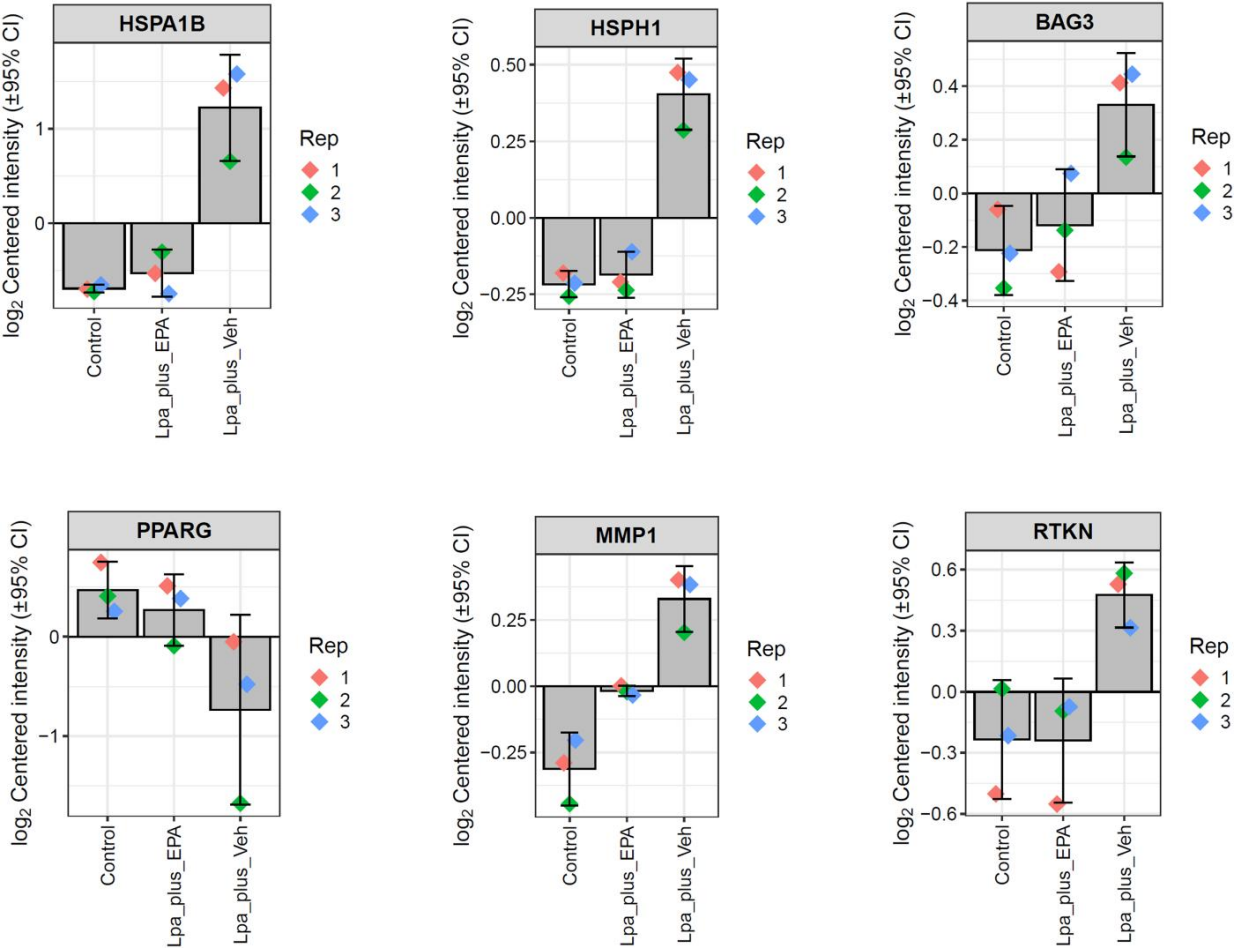
Effects of Lp(a) + vehicle (veh) or EPA and exposed to oxidative conditions on HUVEC protein expression. Bars show raw log2 normalized intensity values of each protein from each treatment groups. Each protein shown here was significantly modulated by Lp(a) + veh, but not Lp(a) + EPA, relative to control (fold change > 1.0 and adj. *P*-value <0.05). BAG3, Bcl-2-associated athanogene 3; HMOX1, haem oxygenase-1; HSPA1B, heat shock protein 70 kDa; HSPH1, heat shock protein 105 kDa; PPARG, peroxisome proliferator-activated receptor gamma; RTKN, rhotekin; SQSTM1, p62.

To probe the impact of EPA-treated Lp(a)-enriched samples on EC function, we employed global proteomic analysis. As seen in the heatmap in Supplementary material online, *Figure S11*, cellular protein expression with Lp(a) + EPA was generally more similar to control than Lp(a) + veh treatment. Further, Lp(a) + veh significantly altered expression of 20 proteins (*Table 1*) relative to control (6 decreased and 14 increased) while Lp(a) + EPA did not significantly modulate expression of any proteins relative to control. The bar plots in *Figure 7* highlight several proteins of interest affected by Lp(a) + veh. There were 1.53- and 3.76-fold increases in heat shock proteins (HSPs) 105/110 and 70 kDa protein 1B, respectively, along with increases in Bcl-2-associated athanogene 3 (BAG3, 1.45-fold), rhotekin (1.61-fold), matrix metalloproteinase-1 (MMP-1, 1.55-fold), haem oxygenase-1 (HO-1, 2.57-fold), and p62 (1.67-fold). There were also 2.62- and 2.13-fold decreases in peroxisome proliferator-activated receptor gamma (PPARγ) and nuclear respiratory factor-1 (NRF-1), respectively.

**Table 1:** Summary of significantly modulated proteins by Lp(a) + veh vs. control

Protein	Fold change	*P*-value
PPARG	−2.62	0.0008
NRF1	−2.13	0.0013
NR1I3	−1.94	0.0012
TPCN1	−1.87	0.0018
RTL9	−1.61	0.0016
P4HA3	−1.53	0.0015
BAG3	1.45	0.0016
HSPH1	1.53	3.35E−05
MMP1	1.55	3.20E−05
RTKN	1.61	0.0008
CAMKK2	1.63	0.0024
SCN1A	1.65	0.0001
SQSTM1	1.67	5.10E−06
IGSF1	1.67	0.0025
ZFAND5	1.77	0.0022
FBXL17	1.82	1.16E−05
RNF214	2.10	1.17E−05
HMOX1	2.57	1.44E−06
ADCY3	2.77	3.04E−05
HSPA1B	3.76	5.62E−07

PPARG, peroxisome proliferator-activated receptor gamma; NR1I3, nuclear receptor Subfamily 1 Group I Member 3; TPCN1, two-pore channel protein 1; RTL9, retrotransposon Gag-like protein 9; P4HA3, prolyl 4-hydroxylase subunit alpha-3; BAG3, Bcl-2-associated athanogene 3; HSPH1, heat shock protein 105 kDa; RTKN, rhotekin; CAMKK2, calcium/calmodulin-dependent protein kinase kinase 2; SCN1A, sodium channel protein type 1 subunit alpha; SQSTM1, p62; IGSF1, immunoglobulin superfamily member 1; ZFAND5, AN1-type zinc finger protein 5; FBXL17, F-box/LRR-repeat protein 17; RNF214, RING finger protein 214; HMOX1, haem oxygenase-1; ADCY3, adenylate cyclase type 3; HSPA1B, heat shock protein 70 kDa.

## 4. Discussion

The key finding of this study was that Lp(a)-enriched plasma simulating levels associated with higher risk (>50% total ApoB) underwent more rapid oxidation than other ApoB particles, including sdLDL. We hypothesized that this results from elevated oxPL content evidenced by higher baseline oxidation levels. EPA inhibited the oxidation of Lp(a) and other enriched lipoprotein fractions in a manner proportional to their oxidation rates in a manner not observed with other therapeutic agents. Lp(a) pre-treated with EPA and then delivered to HUVECs resulted in largely neutral changes in protein expression while Lp(a) pre-treated with vehicle significantly modulated expression of proteins related to cell survival and pro-inflammatory response. The ability of EPA to limit Lp(a) oxidation may contribute to reduced risk of CV events in patients with elevated levels in outcome trials, particularly patients with elevated levels as evaluated in a sub-study of REDUCE-IT.^8^ It may also explain its distinct benefits compared with other n-3FA formulations along with other mechanisms.^20,21^ These results also support characterizing the complete lipoprotein profile for patients as Lp(a)-associated risk remains even when LDL-C is well-controlled.^22^

Controlling for chain length (fixed at C20), fatty acids lacking multiple double bonds or other lipid-lowering agents with distinct chemical properties did not reproduce the potent antioxidant activity of EPA. Oxidative damage to EPA, including its double bonds, strongly correlated with a loss of antioxidant activity proportional to the amount of chemical modification (*ρ* = 1, *P* = 0.0029). This observation is consistent with our molecular hypothesis that the robust antioxidant activity of EPA is dependent on its multiple, methylene-interrupted intact alkene double bonds, as alkenes can stabilize unpaired electrons/free radicals in resonance structures following hydrogen abstraction, especially those held by weaker covalent bonds on bis-allylic carbon atoms (i.e. those found between two alkene bonds) along the acyl chain.^14^ This hypothesis was further supported when deliberate oxidation of the n-3FA ALA led to a similar, consistent loss in antioxidant capacity. When undamaged, ALA exhibited reduced antioxidant activity compared with EPA, possibly arising from the inclusion of fewer unsaturated bonds as has been previously hypothesized.^10^ Probucol, which was developed to interrupt lipoprotein oxidation via free radical scavenging by its diphenol and isopropylidene dithio moieties,^23^ also attenuated lipoprotein oxidation as supported by previous investiagtions^24^ though largely to a lesser extent than EPA. The other lipid-lowering agents investigated in this study have varying effects on Lp(a) levels *in vivo*. Meta-analyses and clinical trials suggest that statins, including rosuvastatin, and fenofibric acid increase Lp(a) levels, though the magnitude and clinical significance remains debatable.^25–28^ Niacin, on the other hand, decreases Lp(a) ∼25%.^29^ Despite these divergent effects on Lp(a) levels, none of the agents exhibited antioxidant capacity in Lp(a)-enriched plasma or the other enriched lipoprotein fractions. Considering there was no change in Lp(a) with IPE treatment, therapeutic modulation of Lp(a) levels does not predict antioxidant activity of that therapeutic agent in this lipoprotein particle.

We have previously shown that EPA possesses greater antioxidant activity than other n-3 fatty acids tested with varying degrees of unsaturation and acyl chain lengths.^10^ That the monounsaturated fatty acid GA exhibited attenuated (but statistically significant) activity suggests that even a single double bond can modify C–H bond strength to allow for hydrogen abstraction and subsequent free radical stabilization. Increasing the number of these double bonds thus could increase antioxidant capacity as shown with ALA in the current study. Counter-intuitively, this relationship does not appear to be linear. Rather, our previous work indicates that DHA (22:6 n-3) has less antioxidant capacity in both lipoproteins and lipid bilayers than EPA despite one more double bond.^10,11,30,31^ Antioxidant capacity of unsaturated fatty acids may therefore depend both on number of double bonds and the length of the acyl chain that influences its stability in the lipid environment.^12,32–34^ Accordingly, in our antioxidant analysis of multiple lipoprotein fractions we limited fatty acids to 20-carbon entities and varied the number of double bonds.

To elucidate further the biochemical basis for the potent antioxidant actions of EPA in these atherogenic lipoproteins, we subjected EPA to spontaneous auto-oxidation over time. By systematically modifying the integrity of EPA double bonds through free radical mediated reactions, we were able to measure the consequences on its antioxidant activity. Specifically, formation of hydrocarbon peroxyl radicals impairs the ability of EPA to stabilize unpaired free electrons in resonance structures. These experiments demonstrated an inverse relationship between the extent of oxidation over time and EPA antioxidant potency. This loss of antioxidant activity correlated directly with increased levels of LOOHs and reactive aldehydes (*ρ* = 1, *P* = 0.0029). Another group used mass spectrometry to characterize the chemical degradation of intact EPA when exposed to oxidative conditions over a similar time frame (4 days).^15^ They found that levels of EPA consistently decrease over time concomitant with increasing levels of oxygenated EPA, including chemical species containing up to six oxygenated moieties. The inclusion of these oxygen-containing adducts diminishes the antioxidant capacity of EPA as evidenced in the current study. This finding again suggests that free radical stabilization requires multiple double bonds, serving as the biochemical basis for inhibition of lipoprotein oxidation regardless of particle size or presence of apo(a). It also highlights the complex nature of EPA as antioxidant as it can serve as both a substrate for oxidation and a quenching agent for the reaction. Routine doses of highly purified EPA are required to replenish the intact molecules and sustain these antioxidant effects over time. This may explain why there was significant event reduction in patients with elevated Lp(a) in REDUCE-IT and in the larger trial cohort treated with 4 g/day IPE.

Additionally, these data provide rationale for the inconsistent CV benefits attributed to low dose (<1 g) fish oil dietary supplements (FODS) containing a mix of n-3FAs, most notably EPA and DHA.^35^ We have previously investigated the chemical integrity of widely used (by sales) FODS.^36^ We found that approximately one-third of these supplements were the essential n-3FAs EPA and DHA, while the remaining fatty acid content comprised a wide range of medium and long chain saturated and unsaturated fatty acids. Because of these low levels, as many as 20 capsules would need to be consumed per day to meet the recommended therapeutic dose in Western populations (4 g/day). Other groups have also found that the actual n-3FA content varies widely between different products from product to product and deviates from advertised levels.^37–41^ Additionally, we detected elevated levels of lipid oxidation species in the products, likely due to the industrial process that render the fish oil vulnerable to uncontrolled high heat and oxygen exposure.^39^ The unsaturated fatty acids, being particularly susceptible to oxidative modification, then lose their biological actions and antioxidant activity.^36^ For patients with elevated Lp(a), FODS would not be expected to fully interrupt particle oxidation and, thus, have little if any impact on associated CV risk.

To investigate the cellular effects of Lp(a) oxidation on protein expression, ECs were challenged with Lp(a) pre-treated with EPA or equivolume vehicle under oxidative conditions. The proteomic results showed that EPA pre-treatment largely neutralized changes in expression caused by Lp(a) oxidation. Lp(a) + veh increased expression of multiple HSPs, namely 70 kDa protein 1B and 105/110 kDa as well as the HSP 70 co-chaperone BAG-3. These three proteins work together to facilitate normal protein folding and degradation of misfolded proteins. Specifically, BAG-3 and HSP 105/110 are considered nucleotide exchange factors which compete for binding to the nucleotide binding domain of HSP 70 with BAG-3 being a stronger competitor than HSP 105/110.^42^ By binding to this domain, these co-chaperones facilitate the release of the protein client bound to the substrate binding domain of HSP 70 and overall diversify the functionality of HSP 70. Members of the HSP 70 kDa family have been elevated in atherosclerotic lesions^43^ and may play contrasting roles in atheroma formation depending on the developmental state (nascent vs. existing) of the lesion.^44^ Oxidized lipoproteins have also been shown to induce HSP 70 expression in isolated human ECs which agrees with our current study. The BAG-3/HSP 70 complex may also promote nuclear factor kappa-light-chain-enhancer of activated B cells (NF-κB) function, thereby providing another potential mechanism for pro-inflammatory cellular responses.^45^

We also observed protein changes which may indicate both pro-inflammatory and pro-atherogenic actions with Lp(a) exposed to oxidation with and without EPA. The significant increase in MMP-1 with Lp(a) + veh may indicate another pro-atherogenic mechanism with oxidized Lp(a), as MMP-1 degrades collagen and weakens the fibrous cap of vulnerable plaques.^46^ Previous studies indicate that oxidized lipoprotein (as oxidized LDL) can increase MMP-1 in ECs.^47^ The nuclear receptor PPARγ, which was decreased with Lp(a) + Veh, plays a key role in endothelial function, with some studies showing that decreased PPARγ activity increases reactive oxygen species and EC dysfunction.^48^ The Rho effector protein rhotekin, also increased with Lp(a) + veh, has various actions including increased NF-κB activation^49^ and, potentially, limiting endothelial nitric oxide synthase activity following RhoA activation.^50^ Both p62 (gene name *SQSTM1*) and HO-1 (gene name *HMOX1*) were increased with Lp(a) + veh, and these proteins work together to facilitate cellular responses to inflammatory signals. The protein p62 promotes expression of *HMOX1* and other genes within the antioxidant response element (ARE) by prompting cytosolic Keap1 to release the transcription factor nuclear factor erythroid 2-related factor 2 (Nrf2) and allows its translocation to the nucleus, where it promotes ARE transcription.^51^ The enzymatic breakdown of haem by HO-1 yields products that promote anti-inflammatory, anti-atherosclerotic, and anti-apoptotic actions.^52^ Previous studies have shown that oxidized lipids—both via phospholipids and lipoproteins—induce HO-1 expression in multiple cell types, including ECs.^53–56^ Finally, NRF-1 plays a crucial role in promoting mitochondrial biogenesis and transcribing electron transfer chain subunits via interaction with peroxisome proliferative-activated receptor gamma coactivator 1α.^57–59^ Decreased expression of NRF-1 with oxidized Lp(a) may indicate loss in normal mitochondrial function. Together, these protein changes indicate that oxidized Lp(a) induces EC stress in a manner not observed when Lp(a) was pre-treated with EPA during oxidation. Thus, EPA not only provides protection for Lp(a) from oxidative damage but in turn can prevent the stress response induced by oxidized Lp(a) in ECs that warrants further investigation.

The relationship between circulating oxidized lipids, including oxPL on ApoB particles, and risk for ischemic events has been observed in the PREVENT Study and a subset of the TNT trial.^6,60^ Moreover, agents that inhibit oxidation such as the active metabolite of atorvastatin were shown to diminish this risk.^61^ Similar to EPA, atorvastatin and an active metabolite (2-*o*-hydroxy atorvastatin) have strong antioxidant scavenging activity in lipoproteins and membranes independent of 3-hydroxy-3-methyl-glutaryl-coenzyme A reductase activity due to a phenoxy group.^62^ This may have contributed to reduced oxPL observed in the TNT trial, along with associated risk. Levels of circulating oxidized LDL are associated with progression of atherosclerosis, especially in patients younger than 60 years.^5^ Beyond their role in lipid oxidation as observed in this study, oxPLs on Lp(a) can stimulate IL-8 production and resultant NF-κB signaling, induce macrophage apoptosis, and promote monocyte penetration through the endothelium and into the arterial wall.^63^ These observations support the role of oxPL in the pro-inflammatory actions of Lp(a).

As mentioned previously, analysis of REDUCE-IT showed significant major adverse cardiovascular events reduction with IPE in patients across the range of baseline Lp(a) concentrations.^8^ Levels of Lp(a) remained unchanged in both trial arms, and approximately 20% of the patients had Lp(a) levels ≥50 mg/dL (125 nmol/L) which is currently regarded as the threshold for ‘high’ ASCVD risk, although this risk is likely better characterized as linear as a function of Lp(a) concentration.^13,64–66^ The clinical benefits of IPE are thus separate from simply reducing Lp(a), standing in contrast to PCSK9 inhibitors which reduce Lp(a) by ∼25% with associated event reduction.^29^ Interestingly, *post-hoc* analyses of the FOURIER and ODYSSEY OUTCOMES trials indicated that the overall event reductions from potent LDL lowering were particularly marked if there was elevated Lp(a).^67,68^ On the other hand, IPE and its active agent EPA elicited significant event reduction irrespective of baseline Lp(a), possibly by interrupting Lp(a) oxidation as shown in the current study. The RNA-based therapies currently in clinical development reduce circulating Lp(a) ∼90% in patients with elevated levels, we await data regarding clinical ASCVD outcomes.^29^ However, the event reduction with these agents may be limited to those individuals with very high levels and risk.

The current study has several limitations. First, we isolated Lp(a)-enriched plasma from a pool of individuals with elevated levels to examine oxidation characteristics, but this is only a model of *in vivo* oxidative conditions. Future work may seek to investigate the effects of oxidized vs. EPA-treated Lp(a) in animals to better understand biological actions on other systems. Additionally, we used a transition metal initiator to accelerate the oxidation reaction, and future studies may seek alternate initiators and models of lipoprotein modification.

Translational perspective We found accelerated lipid oxidation rates for plasma enriched with Lp(a) to levels matching high-risk subjects. Compared with sdLDL-enriched, LDL-enriched, and VLDL-enriched samples, Lp(a)-enriched plasma had significantly accelerated lipoprotein oxidation, possibly due to elevated baseline oxidized phospholipid content. EPA attenuated oxidation of each enrichment in a dose- and time-dependent fashion. EPA-mediated protection of Lp(a) also prevented the expression of many pro-inflammatory proteins in ECs during oxidation compared to Lp(a) alone. The rapid oxidation of Lp(a)-enriched plasma may contribute to enhanced atherogenicity associated with elevated levels. The antioxidant actions of EPA may reduce Lp(a)-associated risk in patients with elevated levels. These data underscore the need to accurately assess the complete lipoprotein profile when determining an individual’s CV risk.

## 5. Conclusions

Lp(a)-enriched lipoproteins (>50% of total ApoB) underwent oxidation more rapidly than sdLDL-enriched and LDL alone. Lp(a) also had higher baseline levels of oxidized lipid, possibly because of inherent oxPL content that may accelerate lipid oxidation. EPA inhibited oxidation in all lipoprotein fractions, and we hypothesize that this antioxidant capacity arises from multiple, intact double bonds which facilitate scavenging activity. Finally, EPA-mediated protection of Lp(a) oxidation largely prevented EC stress responses induced by Lp(a) alone. The more rapid lipoprotein oxidation in Lp(a)-enriched samples may explain, at least in part, increased atherogenicity associated with its elevated levels while the potent antioxidant actions of EPA may reduce the risk of Lp(a) in patients with elevated levels beyond changes in its synthesis, as evidenced in REDUCE-IT. These data also underscore the importance of characterizing the complete lipoprotein profile in accurately assessing an individual’s ASCVD risk.

## Supplementary Material

cvaf144_Supplementary_Data

## Data Availability

The data underlying this article will be shared upon reasonable request to the corresponding author.
