# Supplementary material for: Eicosapentaenoic acid limits the more rapid oxidation of lipoprotein(a) compared with other apolipoprotein B particles
Source: Cardiovasc Res. 2025 Aug 25;121(13):2014–26. doi: 10.1093/cvr/cvaf144 (PMC12560759; doi:10.1093/cvr/cvaf144)
Supplement: cvaf144_Supplementary_Data [file cvaf144_supplementary_data.docx]

**Supplemental Figures.**

**Figure S1. Lp(a)-enriched lipoprotein fractions undergo more rapid oxidation than other lipoprotein fractions.** Data showing values of individual replicates within each treatment group. N = 6 for each group.


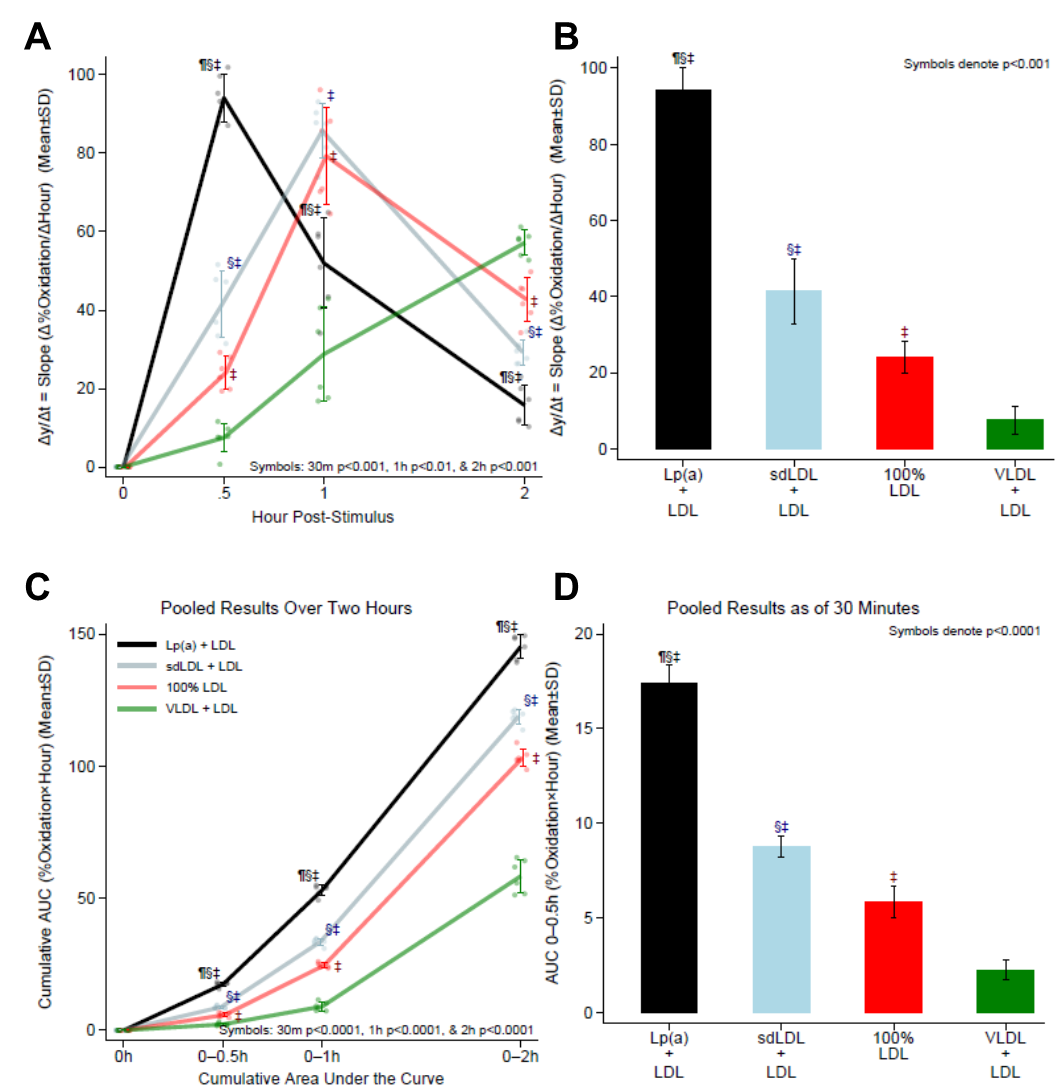


**Figure S2. Lp(a)-enriched plasma exhibited the largest rate of change in percent of total oxidation per change in time after 0.5 h.** Groups are per Figure 1. (A, B) Lines represent the change per unit time, i.e., the slope of the lines in Figure 1, determined as the differences dy/dt for each time point (first derivative of Figure 1). Higher values on the y-axis indicate higher slopes, consistent with more rapid percent oxidation. Notably, the Lp(a) curve achieved the highest slope among all lipoproteins after 0.5 h, consistent with the fastest oxidation rate. (C, D) Cumulative area under the curve by the trapezoidal rule at the intervals shown on the x-axis (i.e., integrating the middle line graph).  Bar graphs for area are for the 0 to 30 minute interval.

**
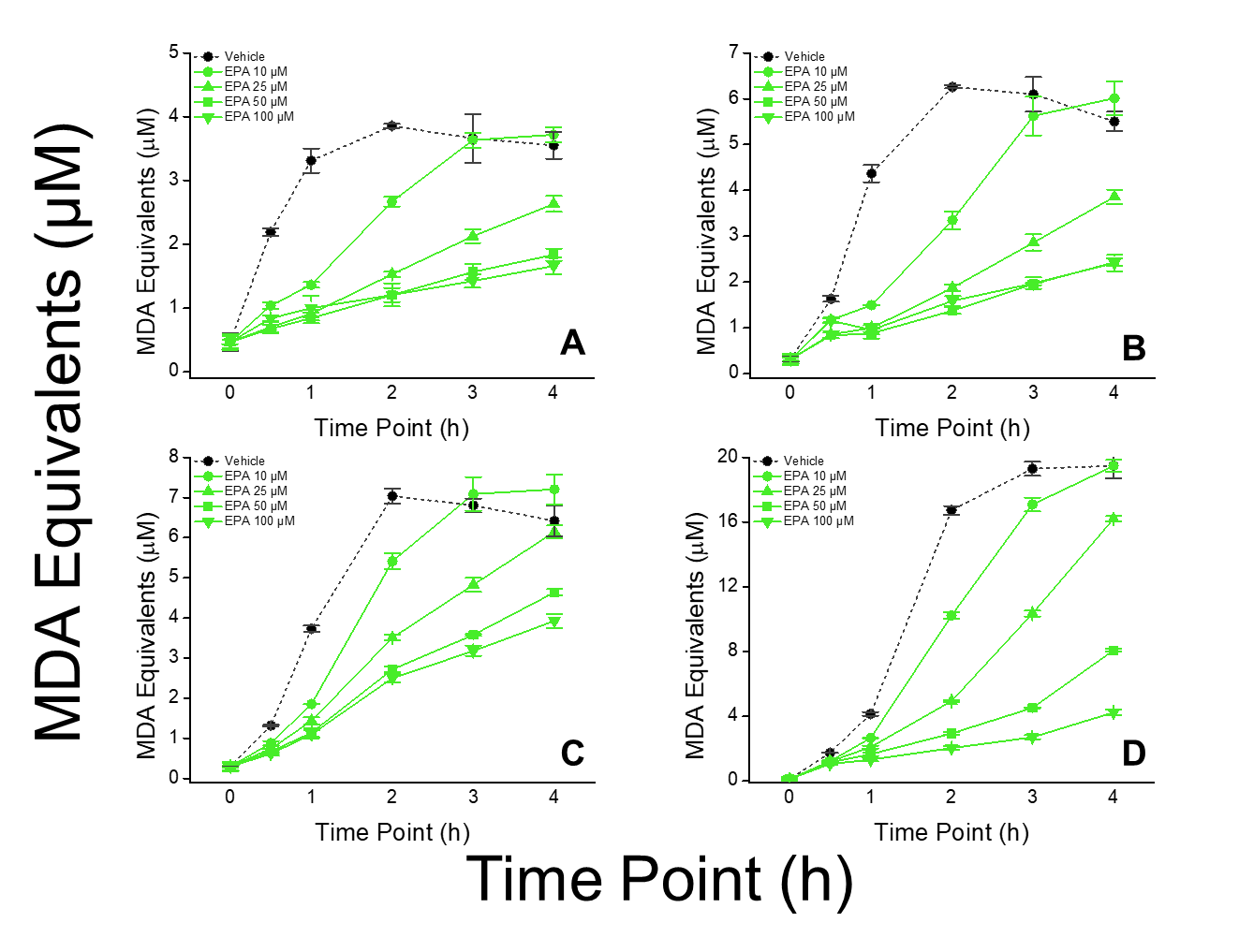
**

**Figure S3. Dose-dependent effects of EPA on oxidation of (A) Lp(a)-, (B) sdLDL-, (C) LDL-, and (D) VLDL-enriched lipoprotein fractions.** Lp(a), sdLDL, and VLDL comprised 66% (by protein) of each respective fraction. Each fraction was incubated in the absence or presence of EPA (10-100 µM). Values are mean ± SD (N = 3).


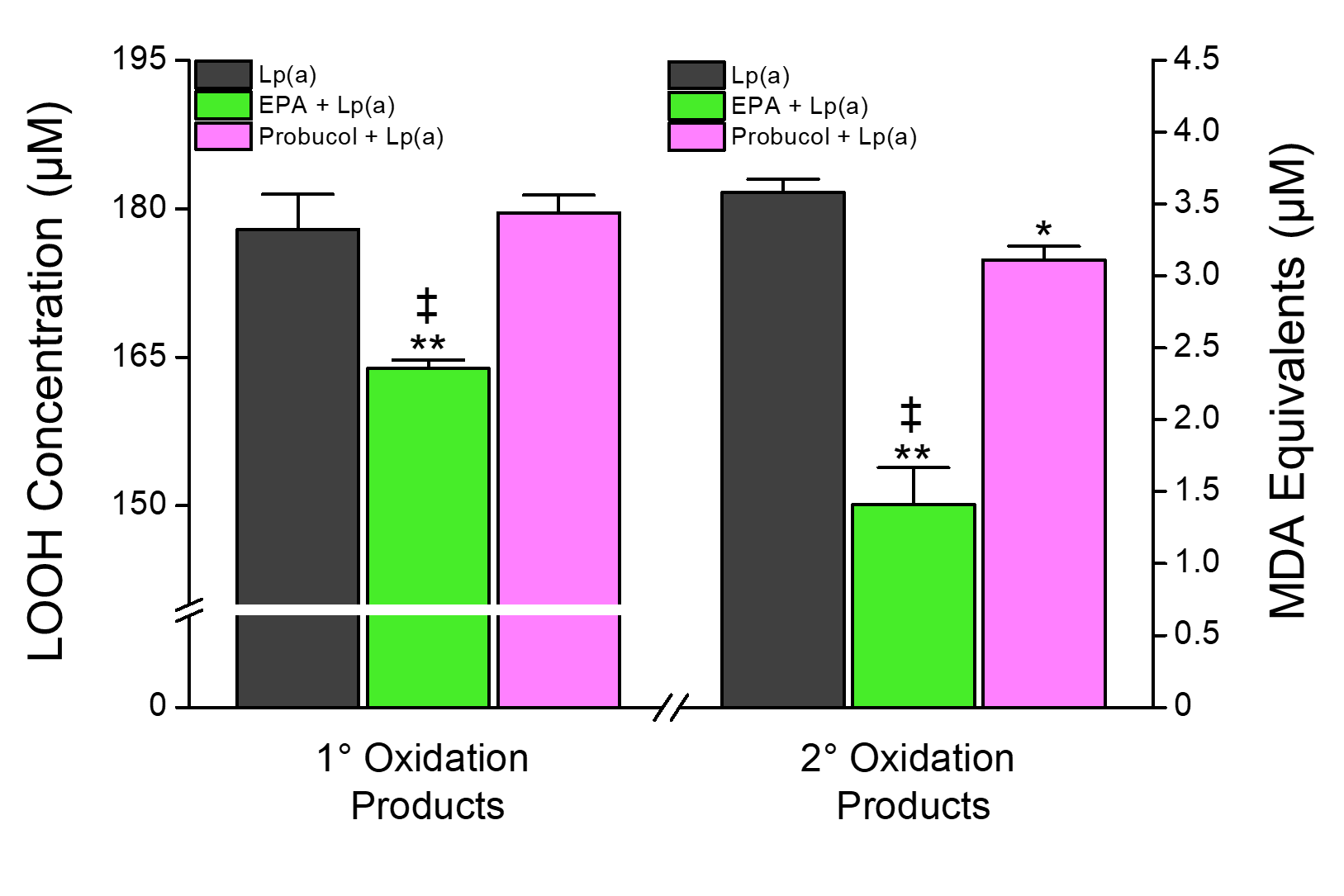


**Figure S4. Correlation of 1° oxidation products (lipid hydroperoxide, LOOH) and 2° oxidation products (malondialdehyde, MDA) from Lp(a) oxidation ± EPA and/or probucol.** Statistical indicators: **p<0.001 versus Lp(a); ^‡^p<0.001 vs probucol (Tukey-Kramer Multiple Comparisons Test; overall ANOVA: p=0.0003). MDA Stats: **p<0.001 versus Lp(a); *p<0.05 vs Lp(a); ^‡^p<0.001 vs probucol (Tukey-Kramer Multiple Comparisons Test; overall ANOVA: p<0.0001). Values are mean ± SD (N = 3).

**
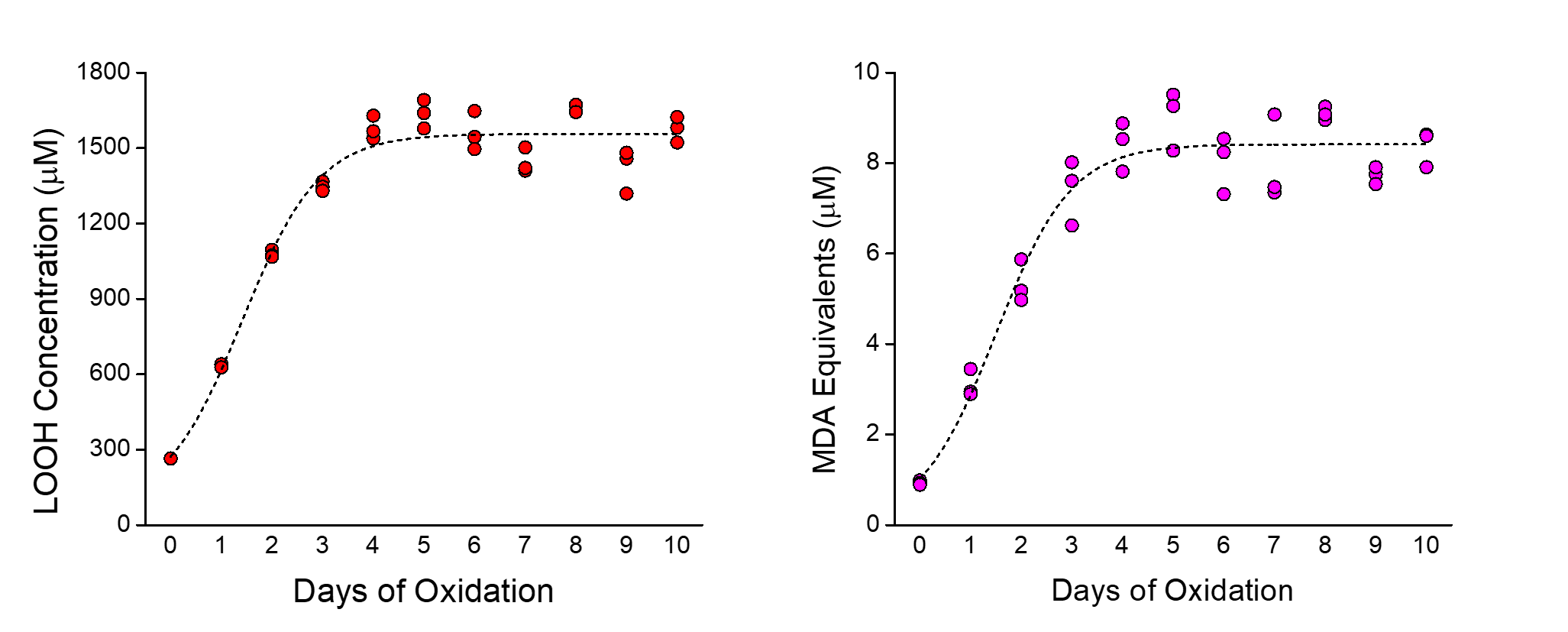
**

**Figure S5. Exposure of EPA to ambient conditions leads to increased primary (LOOH) and secondary (MDA) lipid oxidation products.** Individual aliquots of EPA (10 mg) were exposed to ambient laboratory conditions for 0-10 days. Primary lipid oxidation products detected as lipid hydroperoxides (LOOH). Secondary lipid oxidation products detected as malondialdehyde (MDA). Both products were measured by colorimetric assays. Individual data points from each sample (tested in triplicate) are shown here.


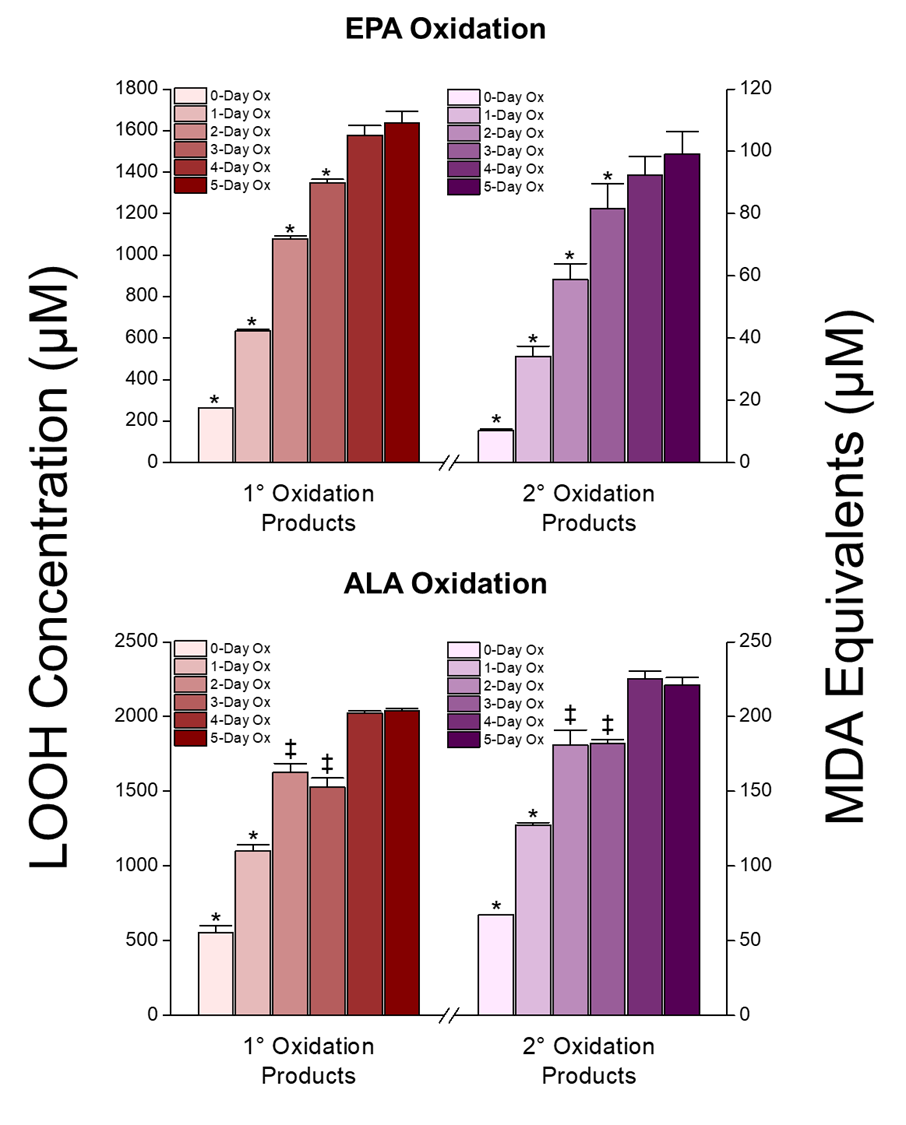


**Figure S6. Exposure of EPA and ALA to ambient conditions leads to increased primary (LOOH) and secondary (MDA) lipid oxidation products thru 5 days.** Statistical indicators: EPA Oxidation - TBARS Stats: *p<0.05 versus all other oxidation durations. LOOH Stats: *p<0.001 versus all other oxidation durations. ALA Oxidation – TBARS Stats: *p<0.001 versus all other oxidation durations; ^‡^p<0.001 vs 4 and 5-day oxidation. LOOH Stats: *p<0.001 versus all other oxidation durations; ^‡^p<0.001 vs 4 and 5-day oxidation (all tests carried out with Tukey-Kramer Multiple Comparisons Test; overall ANOVA: p<0.0001). Values are mean ± SD (N = 3).


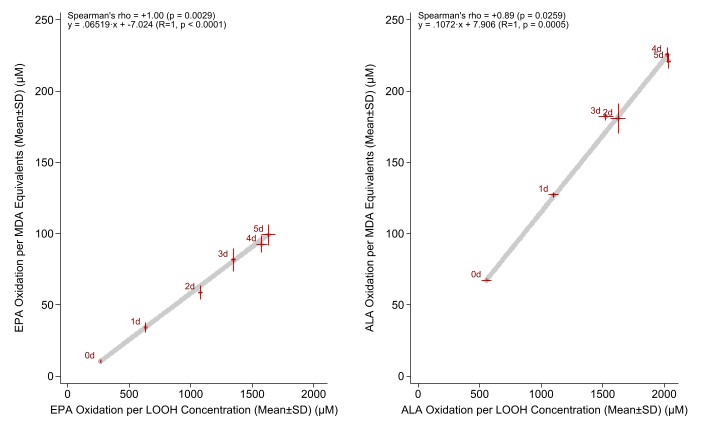


**Figure S7. Correlation analysis of lipid hydroperoxide (LOOH) and malondialdehyde (MDA) formed in oxidized samples of EPA (left) and ALA (right) over time.** The relationship between EPA oxidation and LDL oxidation was monotonic for both measurements of EPA and ALA oxidative damage (LOOH, MDA).


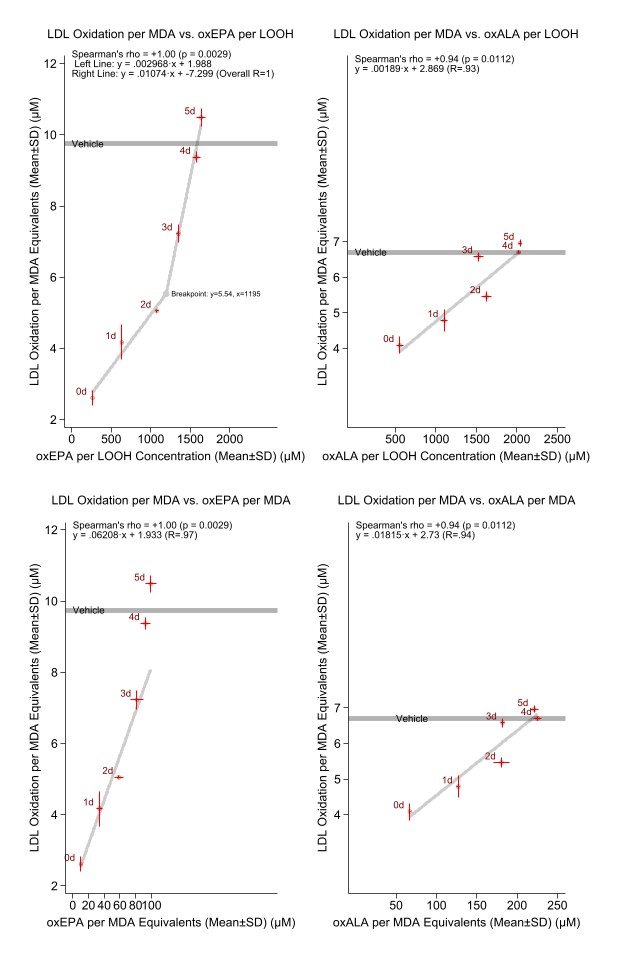


**Figure S8. Correlation analysis of EPA oxidation (LOOH, top left, and MDA, bottom left) and ALA oxidation (LOOH top right, MDA bottom right) with their antioxidant capacity in LDL.** The relationship between EPA/ALA oxidation and LDL oxidation was monotonic for both measurements of n˗3FA oxidative damage (LOOH, MDA). The LDL oxidation data was extracted from the 3-hour time point (see Figure S9).


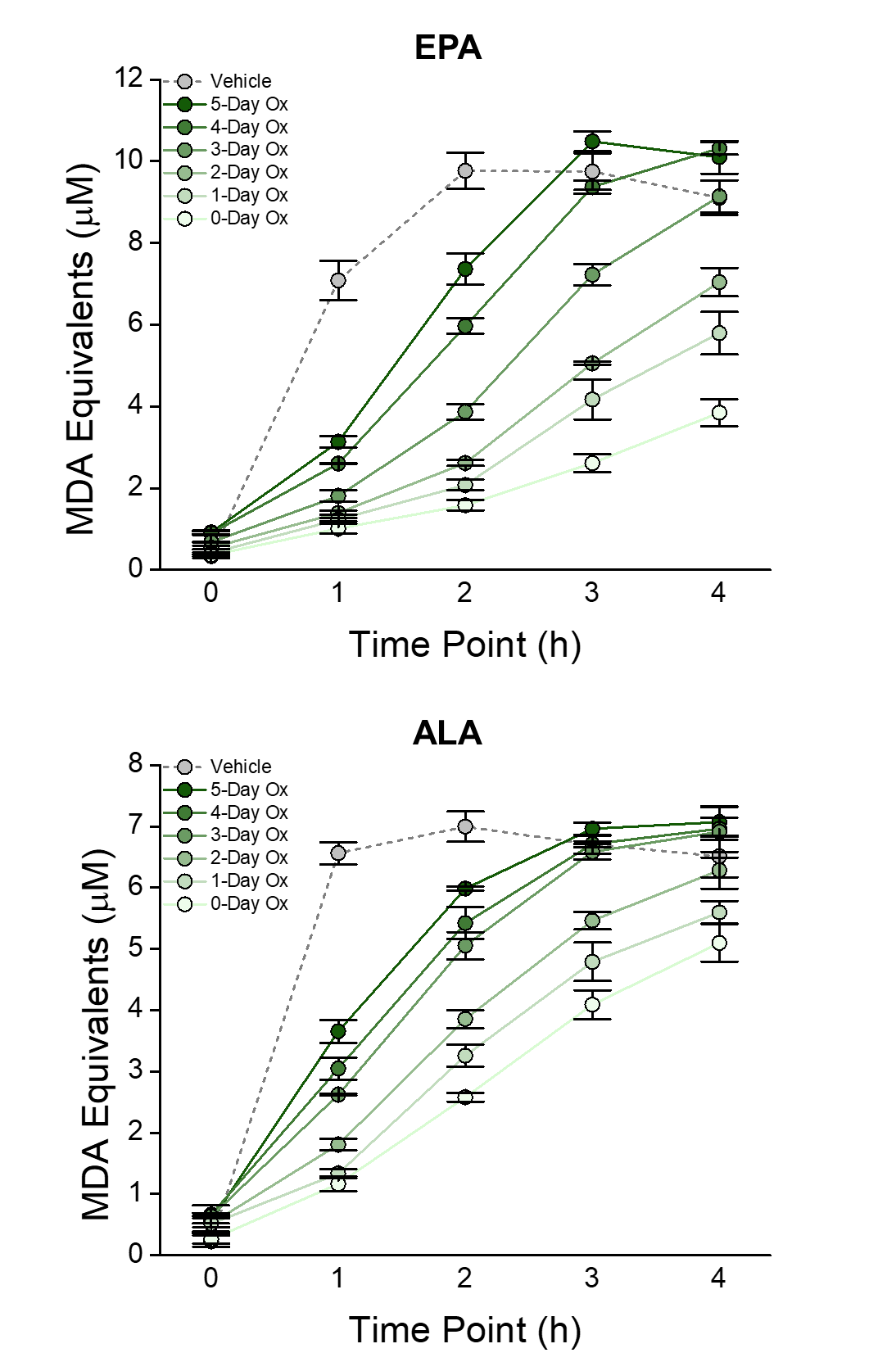


**Figure S9. Exposure of EPA and ALA to ambient conditions decreases antioxidant activity in LDL in a time-dependent manner.** Aliquots of each oxEPA or oxALA sample (50 µM) were incubated with isolated human LDL for 30 minutes. LDL oxidation was then initiated by 20 µM CuSO_4_ and MDA formation was monitored thru 4 hours by colorimetric assay.

**Figure S10. oxEPA and oxALA exhibit decreased antioxidant activity in LDL in a time-dependent manner - % Change vs Vehicle, 3 hr Time Point.** *p<0.01 (Unpaired, two-tailed student's t-test).


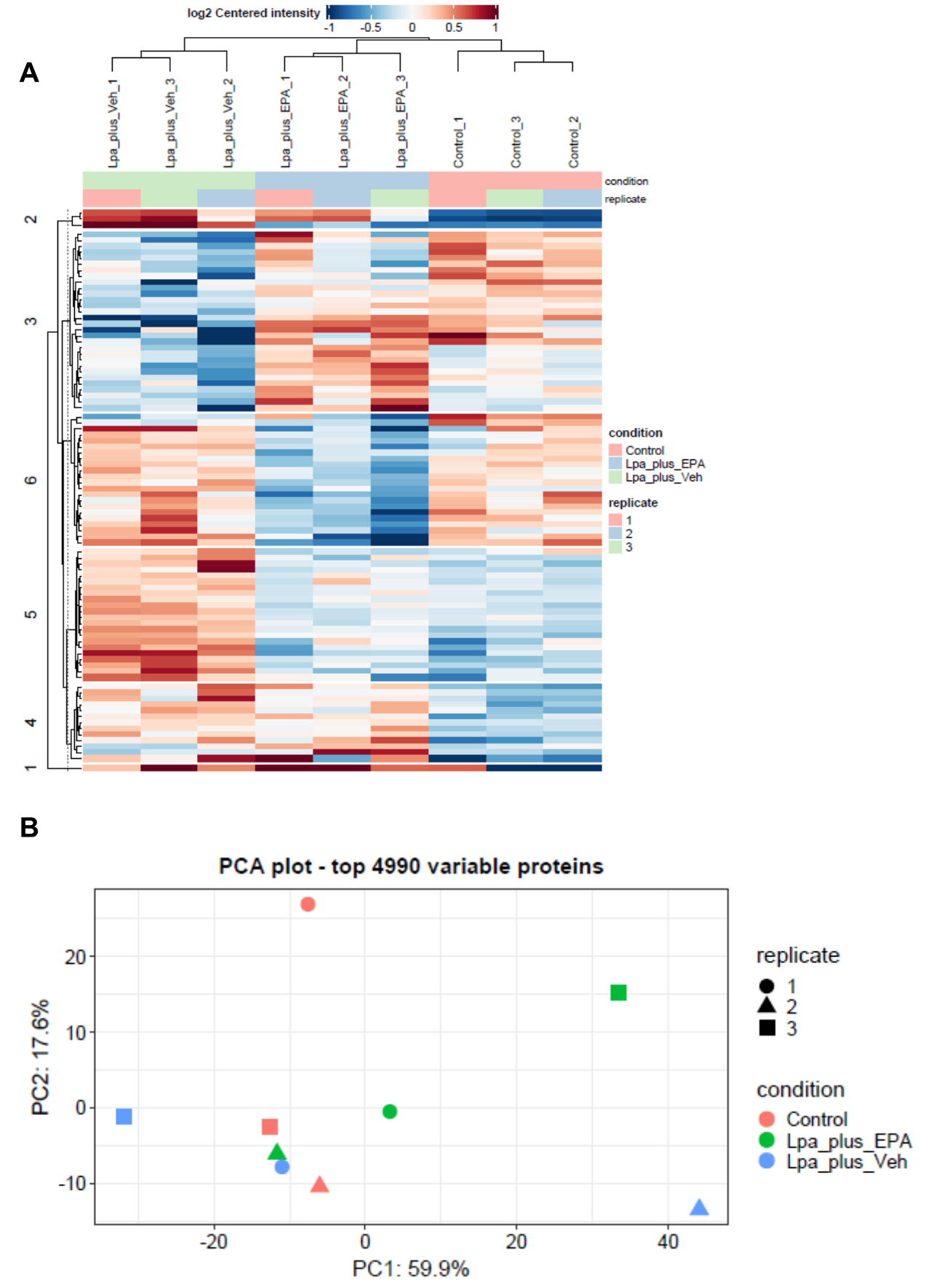


**Figure S11. (A) Heat map of modulated proteins detected in current study and (B) principal component analysis (PCA) plot showing intra- and inter-sample distribution of data set.** In general, there seems to be more similarities between control and Lp(a) + EPA while Lp(a) + Veh lead to differential enrichment of more proteins compared with the other two treatment groups.
